# Neurogenic Inflammation in the Context of Endometriosis—What Do We Know?

**DOI:** 10.3390/ijms222313102

**Published:** 2021-12-03

**Authors:** Renata Voltolini Velho, Eliane Taube, Jalid Sehouli, Sylvia Mechsner

**Affiliations:** 1 Department of Gynecology Charité with Center of Oncological Surgery, Endometriosis Research Center Charité, Campus Virchow-Klinikum, Augustenburger Platz 1, 13353 Berlin, Germany; renata.voltolini-velho@charite.de (R.V.V.); jalid.sehouli@charite.de (J.S.); 2 Institute of Pathology, Charité Universitätsmedizin Berlin, Campus Mitte, Charitéplatz 1, 10117 Berlin, Germany; eliane.taube@charite.de

**Keywords:** endometriosis, neurogenic inflammation, neuroimmune modulation, nerve signalling, peripheral nerve, inflammation, non-hormonal treatment

## Abstract

Endometriosis (EM) is an estrogen-dependent disease characterized by the presence of epithelial, stromal, and smooth muscle cells outside the uterine cavity. It is a chronic and debilitating condition affecting ~10% of women. EM is characterized by infertility and pain, such as dysmenorrhea, chronic pelvic pain, dyspareunia, dysuria, and dyschezia. Although EM was first described in 1860, its aetiology and pathogenesis remain uncertain. Recent evidence demonstrates that the peripheral nervous system plays an important role in the pathophysiology of this disease. Sensory nerves, which surround and innervate endometriotic lesions, not only drive the chronic and debilitating pain associated with EM but also contribute to a growth phenotype by secreting neurotrophic factors and interacting with surrounding immune cells. Here we review the role that peripheral nerves play in driving and maintaining endometriotic lesions. A better understanding of the role of this system, as well as its interactions with immune cells, will unearth novel disease-relevant pathways and targets, providing new therapeutics and better-tailored treatment options.

## 1. Introduction

Endometriosis (EM) is an estrogen-dependent benign, chronic inflammatory disease affecting up to 10 to 15% of reproductive-aged women [1,2,3]. It is associated with a significant societal and economic burden that costs the US economy USD 22 billion annually in lost productivity and direct healthcare costs [3,4,5,6]. The main symptoms of EM include dysmenorrhea, dyspareunia, dyschezia, dysuria, noncyclic chronic pelvic pain, and primary or secondary fertility problems [7,8,9].

**Figure 1:**
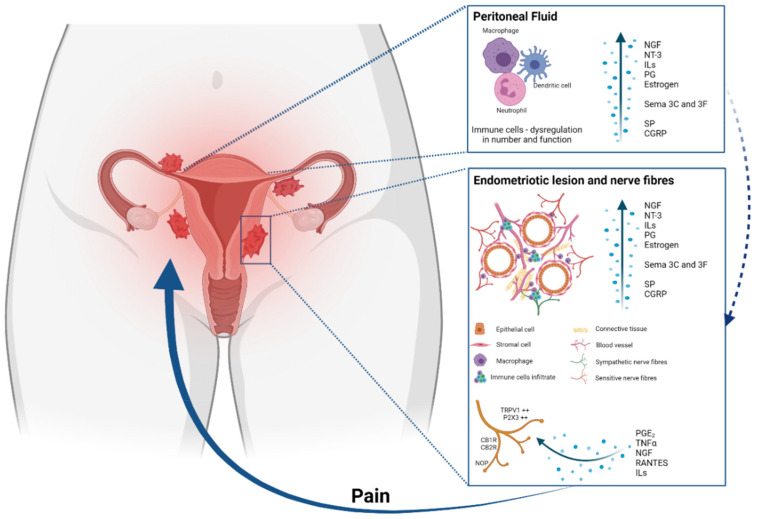
Main pathways involved in the pathogenesis of inflammatory pain in endometriosis. Endometrial fragments in the peritoneum lead to peritoneal inflammation. The same immune response is seen at endometriotic lesions, where the increased production of cytokines, chemokines, growth factors and immune cells also contributes to an enhanced inflammatory environment present in the peritoneal cavity of women with EM. Of these inflammatory mediators, PGE2, tumour necrosis factor-α (TNFα), nerve growth factor (NGF), RANTES and interleukins are also able to stimulate sensory nerve endings and activate a positive feedback loop, further increasing proinflammatory modulator production. The expression of pain receptors is also increased in EM patients’ nerve fibres. The enhanced stimulation and activation of peripheral nerve endings in the peritoneal cavity increase the painful stimuli, initiating and maintaining chronic pelvic pain.

EM is a disease of the uterine tissues (epithelium, stroma, and smooth muscle cells) that leads to ectopic colonization by detachment/desquamation of basal endometrial stem cells during menstruation or by infiltration into the myometrium [10,11,12]. Even if the pathogenesis is unclear, over the last 20 years, our understanding of the mechanisms driving EM lesion growth and pain presentation has evolved (Figure 1). Significant advances have been made in understanding how estrogen drives tissue pathology, resulting in aberrant inflammatory and neuronal states, and promoting the invasion of lesions into the surrounding tissues [13].

In this review, we summarize the role of neurogenic inflammation in endometriotic pain. A better understanding of the role of the peripheral nerve system, as well as its interactions with immune cells, will unearth novel disease-relevant pathways and targets, providing new therapeutics and better-tailored treatment options.

## 2. Patient Presentation, Diagnosis and Current Treatments

There are various symptom patterns and different patient cohorts due to the different kinds of lesions, organs affected, size and diversity of the patient population [14,15,16]. Additionally, non-specific complaints may lead to consultations of various medical disciplines, delaying the diagnosis. An, on average, 10-year-long delay after the onset of symptoms in diagnosis is common [14,15]. After all, more than 60% of those diagnosed with EM report that their complaints started before the age of 20. Furthermore, there is a clear correlation between the duration, the intensity of the complaints, and the extent of the EM manifestations [14,17].

EM remains a clinically suggested diagnosis and is only definitively diagnosed after surgical exploration yields pathologically confirmed EM. Pathologic examination will demonstrate ectopic endometrial-like tissue outside of the uterus containing endometrial epithelium, glands, or stroma, or hemosiderin-laden macrophages (Mφ) [18]. Imaging techniques such as transvaginal sonography and magnetic resonance imaging may be utilized to aid diagnosis but the methodical limitations should be taken into consideration [19,20]. So far, there are no validated biomarkers for diagnosis or therapy monitoring [21].

After diagnosis, EM patients have three classes of treatments available to them: (i) analgesics to manage symptoms, (ii) hormonal therapies designed to inhibit estrogen-dependent growth of lesions, or (iii) surgical ablation/excision of lesions [14]. Beyond medical treatment, many women also find symptom relief during pregnancy/breastfeeding or after menopause [22].

## 3. Pathophysiology of Endometriosis Related Pain

Pain has been defined as a “complex constellation of unpleasant sensory, emotional and cognitive experiences provoked by real or perceived tissue damage and manifested by certain autonomic, psychological, and behavioural reactions” [23]. For the perception of pain, a biochemical signal (1) is converted into a neural signal (2) (sensitization of sensory nerve fibres via activation of the nociceptors). At the spinal level, this signal is modulated (3) and referred (attenuated/amplified) to the brain, where the pain perception occurs (4). Steps one and two are called peripheral sensitization and three and four are central sensitizations [14,16].

If severe EM-associated pelvic pain remains untreated, it will recur monthly. Initially, the pain is perceived cyclically (hormonal-dependent pain) reflecting the classical nociceptive inflammatory pain. If this pain occurs repeatedly, such as monthly, the body’s warning signals take effect, and it is classified as threatening. At this point, the modulation at the spinal level does not regulate it down but rather increases it (hormonal-independent pain). The release of neurotransmitters is altered and several modulating mechanisms are set in motion: the nociceptive field is expanded and EM symptoms such as dysuria and dyschezia may occur [24].

Increasing pain frightens the person experiencing it and makes pain processing more difficult. Severe cramps, accompanied by vegetative reactions, lead the patient to adopt a pain-relieving posture. However, this leads to a reflex contraction of the pelvic floor muscles and eventually to pelvic floor dysfunction. This increases the experienced pain and is known to lead to dyspareunia [25]. Fear of pain during intercourse can strongly influence the ability to relax and the disorder manifests itself. Changes at the central level develop and the patients have an increased risk of developing complex chronic pain syndromes with bladder dysfunction, irritable bowel syndrome, and vulvodynia [24]. This explains the often severe pain that accompanies patients, even in the absence of pathological findings.

## 4. Neurogenic Inflammation

The concept of neurogenic inflammation was first postulated by Bayliss (1901) [26] and stated that peripherally located nerve fibres caused vasodilation in the hind limbs of dogs when stimulated by mechanical, chemical or thermal stimuli. This suggests that afferent fibres could also fire in an antidromic direction. These local antidromic currents were termed the “axon reflex” which is responsible for the vascular flare observed following tissue injury. This activation of sensory neurones leads to an inflammatory response called neurogenic inflammation [26]. This phenomenon is very well investigated in other chronic pain conditions, such as asthma, rheumatoid arthritis, and inflammatory bowel disease, for example. Here, we would like to show the increasing evidence of neurogenically derived inflammatory mechanisms occurring in the EM.

In some EM patients, pain characterization shifts from the more cyclical into an acyclical pattern (see Section 3). In this case, the ongoing activation of sensory nerve fibres releases proinflammatory neuropeptides such as substance P (SP) and calcitonin gene-related peptide (CGRP), both of which are found close to endometrial lesions [6,27]. Furthermore, activation of sensory afferent nerves might initiate the recruitment of mast cells and, subsequently, the release of proinflammatory cytokines as TNFα, NGF, PGE2 and a variety of interleukins, such as IL-1β [28,29]. This inflammation encourages further stimulation of locally circulating mast cells and Mφ [30]. Different studies have shown a high number of these cells in endometriotic lesions as well as an increased amount of proinflammatory cytokines in the peritoneal fluid of EM patients [3,20,21,31,32,33,34]. This contributes to a chronic state of neurogenic inflammation. Increased levels of TNFα and glycodelin correlate with central hyperexcitability in response to repeated electrical stimulation and altered pain response to nociceptive withdrawal reflex [6]. The nociceptive ion channel TRPV1 showed elevated expression on infiltrating adhesions in EM patients, the increase correlating with pain intensity [35,36,37].

Taken together, elevated expression and activation of nociceptors and elevated levels of neuropeptides, other proinflammatory chemicals and cytokines imply that neuroinflammatory processes are present in the central nervous system in EM.

## 5. Neuroimmunomodulation in Endometriosis

### 5.1. Neuro Fibres and Neurotransmitters

Important work over the past years has been performed to understand the mechanisms by which endometriotic lesions induce pain, the primary symptom of patients. Much of this effort has focused on understanding the extent and type of nerves present in endometriotic lesions compared to surrounding tissues. The presence of nerves in endometriotic lesions has been confirmed. In humans, murine models and rat models, ectopic endometrium implants develop sympathetic, parasympathetic, and sensory nerve fibres [24,25,38]. An imbalance in the distribution of sensitive and sympathetic nerve fibres in peritoneal EM lesions in favour of the sensitive nerve fibres of 4:1 has been observed [38]. In the peritoneum of patients without EM, on the other hand, the ratio of sensitive nerve fibres to sympathetic nerve fibres is 1:5. An inverse ratio of the nerve fibres to one another is evident in EM-aged tissue. In line with these data, similar results were also found in intestinal EM [39]. The sympathetic innervation in the intestinal wall, in particular, speaks for influence on the pathogenesis mechanisms, especially since patients with intestinal infection often complain about functional intestinal disorders. The total nerve fibre density is reduced in the periphery of the peritoneal lesion (an area more than 4 mm away from the actual endometrial lesion), most likely due to a reduced number of sympathetic nerves fibres [38].

The neurotransmitters norepinephrine, adenosine, neuropeptide Y (NPY), substance P (SP), vasoactive intestinal peptide (VIP), and endogenous opioids of the different nerve fibre types exert different effects on inflammatory processes by binding to specific receptors of the immune cells. Furthermore, nerve fibres seem to play an immunomodulatory role [40,41]. The expression of neurotransmitter receptors is not evenly distributed among immune cells but appears to be dependent on the microenvironment [42,43,44]. Nerve fibres communicate with immune cells in a synapse-like manner and thus modulate immune cell function [45]. Both efferent (sympathetic and parasympathetic) and afferent (sensitive) nerve fibres influence immune cells through the local release of neurotransmitters [40].

A recent study [46] showed that sensory nerve-derived neuropeptides SP and CGRP facilitate epithelial–mesenchymal transition (EMT), fibroblast-to-myofibroblast transdifferentiation (FMT) and further turn stromal cells into smooth muscle cells (SMCs) in EM, yielding increased collagen production, elevated cellular contractility, and eventually fibrosis. Neutralization of their respective receptors, such as NK1R, RAMP-1 and CRLR, however, abrogates these processes. More remarkably, they showed that lesional nerve fibre density correlated with the lesional expression levels of the receptors, with the extent of lesional fibrosis as well as the severity of pain in EM patients [46]. These data provide strong evidence that sensory nerve fibres play a potent facilitatory role in expediting the development and fibrogenesis of endometriotic lesions. 

More and more data indicate that the peritoneal EM lesions in particular lead to the release of neurotrophic substances into the peritoneal fluid. Significantly increased NGF and NT-3 levels [47] and also estrogen levels [27] have been detected, especially in patients with peritoneal lesions. In vitro analyses using a neuronal growth assay also demonstrated the neuromodulatory properties of EM at the functional level. Peritoneal fluids from patients with and without EM were incubated with sensitive and sympathetic ganglia. The incubation with peritoneal fluid from patients with peritoneal EM showed a significantly increased sprouting of sensitive nerve fibres, but lower sprouting of sympathetic ones. The peritoneal fluid of patients without EM, however, showed the exact opposite: sympathetic nerve fibres were induced, while sensitive nerve fibres were inhibited. This reflects the results of the changes associated with peritoneal EM and demonstrates serious changes in peritoneal innervation, which are caused by EM, lead to far-reaching changes in the entire peritoneal milieus, and may be the cause of the complex symptoms of the patients [48]. Interactions between endometrial lesions, nerve fibres, and immune cells are considered to be essential factors in these changes.

### 5.2. Semaphorins and Neuromodulation

The semaphorin family of proteins includes many secreted and membrane-associated proteins. There are approximately 20 distinct members in higher vertebrates, and all contain the family’s signature semaphorin domain, a ~500 amino acid N-terminal that is the key extracellular signalling domain of these proteins. Semaphorins act as nerverepulsive factors that become extracellularly effective through a specific repulsive influence on either sympathetic or primary afferent sensitive fibres through various surface receptors, neuropilin-1 (Nrp1), and neuropilin-2 (Nrp2) [49,50].

In normal human endometrial tissue, the expression of many semaphorins is upregulated in the proliferative stage of the menstrual cycle, when estrogen is at its highest [39]. Estrogen has been found to induce the expression of semaphorins in uterine tissue [51]. Semaphorins 3C and 3F (Sema 3C and Sema 3F, respectively), in particular, are known as nerve repellent factors and are upregulated in EM-associated Mφ in rat and mouse models [51,52]. In women with EM, studies revealed an affected innervation and a significant increase of Sema 3C and 3F and their receptors in peritoneal endometriotic tissue. Thereby, the expression of the receptors was identified on the membrane of noradrenergic nerve fibres and vessels. Mφ and activated fibroblasts were found in higher density levels and additionally express semaphorins in peritoneal endometriotic tissue. Inflammation leads to an increased release of immune cells, which secrete a variety of inflammatory factors capable of affecting innervation. Therefore, these data suggest that the chronic inflammatory condition in EM might contribute to the increase of semaphorins, which could affect the innervation in peritoneal EM [53,54].

## 6. Pain Receptors in Endometriosis-Associated Nerve Fibres

The understanding of pain generation, as this is the main problem in EM patients, is of great importance: the ectopic lesions themselves release pain mediators and activate nociceptors (transduction and transmission). In most cases, this is strongly estrogen-dependent and leads to cyclical nociceptive pain (dysmenorrhoea and cyclical pelvic pain), which is treated by hormones or non-steroidal antiphlogistics (NSAP). However, with ongoing disease, the cyclical pain characterization shifts into an acyclical chronic pain (pain development under hormonal treatment, NSDAP resistant pain, increasing pain severity) [55,56]. We analyzed this phenomenon in nearly 100 patients with acyclical pain under hormonal treatment and found it in 100% of the peritoneal ectopic lesions with extended inflammatory reactions [16]. This phenomenon might especially be due to neurogenic inflammation and activation of peripheral sensory nerve fibres. The peripheral sensitization seems to be stressed and for this, the development of non-hormonal anti-inflammatory compounds is of great interest. So, besides the understanding of the inflammatory reaction, the peripheral sensitization of nerve fibres is also of great importance.

### 6.1. Purinergic Receptors

In acute and chronic pain models, small- and medium-diameter sensory neurons, which express transient receptor vanilloid-1 (TRPV1) channels and/or adenosine triphosphate (ATP)-gated P2X3 receptors, are the important pain transducers of noxious stimuli [57]. In women with EM, TRPV1 receptor expression has been demonstrated to be elevated in endometriotic lesions and correlated with pain [36,37,57].

Purinergic receptors are ligand-gated ion channels that are expressed in sensory neurons which can be activated by adenosine triphosphate (ATP). P2X3 is one such purinergic receptor. In women with EM, ATP released during retrograde menstruation and due to mechanical stretch from endometriotic adhesions or fibrotic scar tissue could potentially activate P2X3 receptors, leading to neuronal hypersensitivity and pain [58]. Indeed, its expression in the endometrium of women with EM was significantly higher compared to control endometrium and interestingly was also correlated with the severity of pain reported [58,59]. Current interest in targeting P2X3 in EM is high, with multiple pharmaceutical companies having initiated drug programs, one of which has begun to enter Phase IIb clinical trials (NCT04614246).

### 6.2. Opioid Receptors

The endogenous opioid peptides (EOPs) are derived from proopiomelanocortin (POMC), proenkephalin (PENK), and prodynorphin (PDYN) precursors and exert their effects by binding to the G-protein-coupled receptors δ-opioid receptor (DOR), κ-opioid receptor (KOR), μ-opioid receptor (MOR), and nociceptin/orphanin FQ opioid peptide receptor (NOP) [59,60]. The peripheral opioid system plays a crucial role in inflammatory reactions and neurogenic inflammation since this system is activated and modulated by inflammation. During this process, analgetic factors such as anti-inflammatory cytokines and opioid peptides are released, promoting antinociceptive actions. In chronic inflammation, the release of anti-inflammatory mediators and opioid peptides is decreased, improving the perpetuation of inflammation. Since EM is a chronic inflammatory disease with a disturbing pain mediation and analgesia, disruption in the expression of opioid peptides or opioid receptors might be involved in the inflammatory condition and pain pathogenesis in this disease and alterations in the expression of those receptors and their respective ligands should be urgently investigated. Indeed, the mRNA of three opioid peptide precursors has been described in the endometrium [61]. Finally, DOR and KOR have been described in Ishikawa human endometrial cells, while MOR is absent [62,63]; in fact, MOR has been localized only in the uterine luminal epithelium cells of the pregnant mouse [64] and in EM stromal cells [65,66,67]. NOP has not been tested. The proven presence of most of the compounds of the opioid system in the endometrium denotes the role of this system in processes occurring in the endometrium, including EM.

### 6.3. Endocannabinoid Receptors

The first biologically active component of cannabis was identified in the 1960s as delta-9-tetrahydrocannabinol (THC), a potent drug classified as a sedative-hypnotic. This was followed by the discovery of two cannabinoid receptors, cannabinoid receptor 1 (CB1R) and cannabinoid receptor 2 (CB2R), in the early 1990s. Both are G-protein-coupled receptors and serve as the primary sites of action for THC. Together, they are involved in the major neuromodulatory endocannabinoid system (ECS), whose primary goal is to promote homeostasis [68]. The ECS is widespread, and CB1Rs are found throughout the central nervous system and some peripheral tissues, while CB2Rs are primarily found in peripheral tissues and immune cells [69]. Cannabinoids act on receptors other than CB1R and CB1R. They modulate transient receptor potential (TRP) channels, including TRP vanilloid (TRPV) channels, which are involved in neuropathic pain signals in EM [70]. 

There is a broad range of mechanisms by which cannabinoids modulate pain, and given the high impact of EM on women’s quality of life such as the high prevalence of EM, cannabinoid-mediated effects on EM-related pain have become a subject of inquiry [68,69].

Different categories of EM pain may be modulated by cannabinoids [71,72]. There is evidence that a disruption of the normal ECS potentiates pain in EM patients. In women with EM, decreased CB1R expression compared to controls and increased anandamide (AEA) and 2-arachidonoylglycerol (2-AG) expression, both endogenous ligands of the CB1R and CB2R, are consistent with a negative feedback loop that is permissive of inflammation [68].

In vitro studies have shown that exogenous cannabinoids could correct these dysregulations. When endometrial cells were treated with cannabinoid agonist WIN 5512-2, the result was decreased cell proliferation, decreased reactive oxygen species production, and reduction in alpha-smooth muscle actin expression, lending supporting evidence to the anti-inflammatory effects of cannabinoids [73]. Bilgic et al. [74] also found that CB1R and CB2R expressions are decreased in EM tissue compared to control, with concurrently decreased apoptosis indexes. The same study found that exposure of EM tissue to CB1R and CB2 agonists resulted in pro-apoptotic effects. However, animal model studies have shown divergent results. A nude mouse model of transplanted human deep infiltrating EM confirmed the antiproliferative effects of cannabinoids on the growth of deep infiltrating lesions [64]. In contrast, in a mouse analogue of early-stage EM, the activation resulted in an increased disease burden [75]. The complexity of the ECS superimposed on the two different mouse models used may account for the different results [76].

Endocannabinoids have also been shown to trigger endometrial cell migration [77]. Moreover, CB1Rs have been found on the sensory and sympathetic neurons innervating EM lesions [78]. Given the above role of the ECS in the EM modulation, cannabinoids have been proposed as a putative therapy. In fact, an Australian cross-sectional survey showed that self-management strategies were very common in EM patients and that cannabis and cannabis products were among the most effective at pain reduction [79].

## 7. Endometriosis and Inflammation

Menstruation is an inflammatory process characterized by an increase in a variety of tissue-resident immune cells. A complex interaction between resident immune cells and uterine stromal cells modulates the biosynthesis and release of pro-inflammatory cytokines, chemokines, and prostaglandins (PGs), resulting in local vasoconstriction [3,6]. When retrograde menstruation occurs, endometrial fragments adhere and form lesions within the peritoneum. During this process, inflammatory cells are recruited to the lesions. This immune response is evident at lesion sites, with increased inflammatory cytokines/chemokines, growth factors, neutrophils and PGs found within the peritoneal cavity of EM patients [3,6,20,80,81].

Since EM is considered a chronic inflammatory disorder, the neuromodulatory mechanisms of the EM-associated immune cell infiltrate (EMaICI) need to be considered. In all investigated types of EM, immune cell infiltrates were observed and characterized as a mixture of several immunocompetent cells (T cells, B cells, and Mφ) [31,82,83]. In endometriotic lesions and also in eutopic endometrial tissue of EM patients, the numbers of EMaICI were significantly higher than in control tissue and seem to be associated with a chronic inflammatory process [84]. EMaICI were observed and characterized in all the types of EM by our group as T lymphocytes (CD3+), helper T lymphocytes (CD4+), cytotoxic T lymphocytes (CD8+), antigen-experienced T lymphocytes “memory cells” (CD45RO+), macrophages (CD68+), and B lymphocytes (CD20+) [85]. The characterization of various types of immunocompetent cells in EMaICI demonstrated several distinct immunological reactions within the microenvironment of different endometriotic lesions.

Dendritic cells (DC), Mφ, mast cells (MC) and neutrophils play a central role in chronic inflammatory diseases [32,86,87]. Under normal conditions, immature DCs mature and travel to the lymph nodes in response to foreign antigens or other inflammatory signals, where the antigens are presented to T cells. However, the maturation of DCs declines in EM, with it being hypothesized that immunological components form part of the antigen capture and/or presentation activity. This process might be altered in the endometrium of women with EM. Consequently, EM-circulating anti-endometrial antibodies could mask endometrial antigens. As a result, endometrial antigens might not be effectively recognized, with lost endometrial fragments remaining, potentially leading to ectopic establishment [83,88,89].

Mφ are abundantly recruited to lesions of EM after activation by certain chemokines and cytokines. These cells can release different types of inflammatory substances, creating an inflammatory microenvironment that contributes to the establishment and growth of endometriotic lesions. In turn, these changes can induce the Mφ recruitment and as a result, form a vicious circle during the development of the disease [3]. Mφ are classified as M1 Mφ, which exhibit proinflammatory activity, and M2 Mφ, which provide an anti-inflammatory environment and are capable of remodelling tissue through pro-fibrotic activity. In the context of EM, the differentiation between M1 and M2 Mφ seems to be shifted in favour of M2 in EM and this is particularly important since M2 Mφ are more immunologically tolerant [20,30,90]. It has been reported that endogenous Mφ are involved in tissue remodelling during the development of EM, and the M2 Mφ, in particular, is required for the growth of ectopic lesions in a mouse model [30]. More recently, two independent groups found a decrease in the percentage of M1 and an increase in M2 Mφ in peritoneal washings of EM patients, especially those with advanced disease (stages III–IV) [91,92].

MCs are known to be key players of the immune system, especially during allergic reactions. However, increasing evidence supports the involvement of these cells also in the inflammatory process of EM. High numbers of degranulated MC have been found in endometriotic lesions showing their influence on EM lesions in development, survival, phenotype, and function via the regulation of other immune cells (monocytes/Mφ, granulocytes, DC, and T-B lymphocytes) [31,32,33,34,93,94].

Neutrophils are considered simple foot soldiers of the innate immune system and are undoubtedly the major effectors of acute inflammation. Several lines of evidence indicate that they also contribute to chronic inflammatory conditions as well as adaptive immune responses [95]. The infiltration of neutrophils into the peritoneal cavity is significantly increased in EM patients compared with that in healthy women, especially in the early stage of EM [96,97]. In EM, neutrophils in the abdominal cavity can secrete an effective pro-angiogenic factor, VEGF, which is also increased in the PF in the EM. As a result, neutrophils may support the growth of endometriotic lesions by secreting VEGF. Moreover, there may be some other nonclassical factors secreted by neutrophils that can promote inflammation and neovascularization in EM [97].

Aberrant expression of several cytokines by inflammatory cells, such as IL-1, IL-4, IL-6, IL-8, IL-10, IL-33, TNFα and growth factors, e.g., transforming growth factor (TGF-β), insulin-like growth factor (IGF-1), hepatocyte growth factor (HGF), epidermal growth factor (EGF), platelet-derived growth factor (PDGF), and vascular endothelial growth factor (VEGF), have been reported in EM [89,98]. Indeed, cytokines such as IL-8 and TNF-α are known to promote endometrial cell proliferation, endometrial adhesion, and angiogenesis. Furthermore, endometriotic lesions can induce the expression of PGs, MCP1, glycodelin, and other inflammatory mediators [25,99]. Specifically, PGE2, PGF2α, and TNF-α are produced and increased in the early stage; TNF-α, NGF, and IL-17 can cause persistent inflammation; and PGE2, PGF2α, transforming growth factor-β (TGF-β), glycodelin, and TNF-α can induce the sensation of pain [3,25,100]. A recent study showed that cytokine analysis of PFs could differentiate women diagnosed and stratified laparoscopically with ovarian endometrioma, peritoneal, or deep infiltrating EM. This suggests that certain cytokine signatures could be driving different biological signalling events and immune responses in these patients [101]. These inflammation-associated substances act on inflammatory cells in turn. These retroactions lead to more inflammatory cell recruitment in lesions with a subsequent alteration in the original peritoneal and pelvic environments and the formation of a new inflammatory microenvironment. The growth, implantation, infiltration, and migration of EM lesions occur subsequently and retroact on inflammatory cells and substances. This vicious cycle contributes to the aggregation of EM-associated inflammation [3].

## 8. Conclusions

Even if the pathophysiology of EM is not completely understood, it is well established that the immune system plays a key role in this disease. Moreover, the peripheral and central nervous systems are intimately involved in EM disease and symptomatology. Nociceptor neurons possess many of the same molecular recognition pathways for danger as immune cells and in response to danger, the peripheral nervous system directly communicates with the immune system, forming an integrated protective mechanism. The dense innervation network of sensory and autonomic fibres in peripheral tissues and high speed of neural transduction allows for rapid local and systemic neurogenic modulation of immunity. Peripheral neurons also appear to play a significant role in immune dysfunction in autoimmune and allergic diseases. The literature provides evidence for an overall heterogeneity in EM, rather than a standard approach. This strongly suggests a personalized treatment approach based on aetiology and symptomatology. Current treatments, both pharmacological and surgical, are addressed at providing symptom relief and are mainly focused on complex pain management, without effective results for all patients. Several studies are attempting to overcome these obstacles by identifying molecular targets to develop new therapeutic approaches to improve the quality of life of affected women. Once we put aside the paradigm of lesion-specific and cyclical inflammatory pain, further areas will open up and increase the treatment opportunities in a multimodal approach.
